# Can companies get more government subsidies through improving their ESG performance? Empirical evidence from China

**DOI:** 10.1371/journal.pone.0292355

**Published:** 2023-10-03

**Authors:** Xuan Zhang, Jingxian Zhang, Yongjie Feng

**Affiliations:** 1 Business School, Northwest Normal University, Lanzhou, China; 2 School of Economics and Management, University of Science and Technology Beijing, Beijing, China; Usak University: Usak Universitesi, TURKEY

## Abstract

Environmental protection and social obligation fulfillment have become hot subjects as the "dual carbon" approach has been developed and deepened. The ESG system is consistent with China’s current policies, abandoning the traditional business philosophy of economic supremacy in favor of comprehensively measuring corporate social responsibility and sustainable development capability across three dimensions: environmental (E), social (S), and corporate governance (G), which receive widespread attention from all sectors of society. Based on observational data from A-share listed businesses in Shanghai and Shenzhen from 2011 to 2020, this study empirically evaluates the influence and mechanism of ESG on government subsidies. The research results indicate that enterprises can receive more government subsidies by improving ESG performance. Mechanism analysis found that corporate transparency plays a positive mediating role in the process of ESG affecting government subsidies. Further research on political affiliation and property rights has found that companies without political affiliation are more inclined to receive more government subsidies by improving ESG performance, and the impact of political affiliation and ESG performance on government subsidies is mutually complementary. Enterprises with different property rights have different strengths of motivation to increase government subsidies by improving ESG performance. State owned enterprises (excluding central enterprises) are the strongest, followed by non-state-owned enterprises, and central enterprises are the weakest. Therefore, enterprises should be further encouraged to strengthen ESG construction, improve the quality of ESG information disclosure, improve resource allocation efficiency, and promote high-quality development of enterprises.

## 1. Introduction

In recent years, environmental problems, including resource misuse, pollution, and climate change, have received a lot of attention. In 2020, China proposed the "dual carbon" goal at the 75th UN General Assembly, clearly propose to achieve the goals of "carbon peaking" by 2030 and "carbon neutrality" by 2060. In addition, the report of the 20th Party Congress in 2022 emphasized the necessity of respecting, conforming to, and protecting nature as a precondition for the overall construction of a socialist state and called for developing a blueprint that would allow humans and nature to coexist in harmony [1]. While the nation actively encourages environmental conservation, economic development has also reached a certain level. The Communist Party of China’s 19th National Congress report pointed out that China’s economy has transitioned from high-speed growth to high-quality growth. It is essential to actively promote the sustainable development of enterprises in order to keep up with the tide of the economic revolution [2]. The ESG system is consistent with China’s "dual carbon" and high-quality economic development, and it has garnered considerable societal attention. The ESG system denies the traditional business philosophy of prioritizing economic efficiency, and measures the degree of corporate social responsibility fulfillment and sustainable development ability from three aspects: environmental, social, and governance. It is in line with the background of China’s "dual carbon" and high-quality economic development, and has received widespread attention from society. The United Nations Social Responsibility Investment Principles (UN-PRI) first proposed the ESG concept in 2006, urging enterprises to balance economic and social benefits and encouraging the incorporation of ESG concepts into business investment decisions, thereby providing important insights for promoting economic development [3]. The early ESG notion was business ethics investment, which subsequently evolved into social responsibility investment and then into sustainable investing, which is a sublimation of conventional corporate social responsibility. ESG rejects the traditional business philosophy of prioritizing economic efficiency in favor of measuring the degree of corporate social responsibility fulfillment and the level of sustainable development of enterprises from three perspectives: environmental (E), social (S), and corporate governance (G), which is in line with China’s implementation of the "dual carbon" goal and high-quality economic development.

Enterprises encounter several challenges throughout the economic transition process, such as restrictive corporate structures, insufficient finance, reputation harm, and so on. Among these, adequate and stable capital flow is not only an essential measure of financial health but also the cornerstone for firm survival and growth. As a result, funding is critical in the process of company transformation and upgrading. According to the theory of resource reliance, the survival and development of businesses are dependent on both internal and external causes. As an important external stakeholder, the government has high-quality resources. It offers targeted firms with financial subsidies, tax breaks, technical innovation, and other forms of assistance by selecting enterprises that satisfy projected financing conditions. This promotes enterprise transformation and upgrading. Existing research shows that government subsidies can improve enterprises’ environmental performance by enhancing innovation in green processes [4], incentive enterprises to increase investment in research and development [5], and promoting enterprises to raise productivity [6]. Scholars have pointed out that one of the ways enterprises can get government aid is by carrying out their social responsibility obligations [7], and that doing so helps enterprises keep their political connections strong and thus get more aid from the government [8]. The ESG system, which has some economic significance and practical value, thoroughly assesses the performance of corporate social responsibility from three perspectives: environment, society, and corporate governance. The academic community currently holds a variety of opinions on the potential role that ESG can play. Most scholars believe that the ESG system can improve corporate financial reporting’s transparency and reduce the issue of information asymmetry between trading parties [9]. Enterprises with good ESG performance release positive signals to the outside world by improving transparency, thereby shaping a good external image [10], building reputation effects, and reducing operational risks. A good business environment is conducive to reducing audit costs [11], improving financial performance [12, 13], and increasing enterprise value [14–16], thereby promoting sustainable development of the enterprise [17]. Because adopting the ESG idea unavoidably consumes limited resources inside the organization and has a financial impact, some academics have a negative view of the economic repercussions of ESG. They believe that it is difficult for enterprises to balance economic and social benefits at the same time, and improving ESG performance will have a negative impact on enterprise performance [18, 19]. In addition, a few studies indicate an unclear relationship between ESG performance and corporate performance [20]. From the data above, it is clear that current research focuses more on the internal advantages of businesses and investigates the economic effects of ESG. There isn’t much research studying the effect of ESG on government subsidies that link enterprises and the government from an outside perspective. This essay does extensive research on this subject to address the inadequacies of previous studies.

This article investigates the practical question of whether ESG can encourage the growth of government subsidies based on the policy background of "dual carbon" and high-quality economic development. This is helpful for further unleashing the economic consequences of ESG and giving enterprises a strong grip to increase the level of government subsidies. It has theoretical worth and practical significance, in addition to partially filling up research gaps. In view of this, this article takes government subsidies as the entry point, selects ESG data from Huazheng Index Information Service Co., Ltd. (hereinafter referred to as "Huazheng"), which currently has the most comprehensive coverage and fastest update, and uses observational data of A-share listed companies in Shanghai and Shenzhen from 2011 to 2020 to empirically examine the impact of ESG performance on government subsidies. Research has found that good ESG performance can enhance the level of government subsidies. Corporate transparency plays a positive mediating role in the process of ESG affecting government subsidies. A moderating factor that influences the impact of ESG on government subsidies is political connection. According to research, the influence of political connection and ESG on government subsidies is complementary, and enterprises without a political connection are more likely to increase their government subsidies by enhancing their ESG performance. Further analysis reveals that enterprises with different property rights have different strengths of motivation to increase government subsidies by improving ESG performance. The strongest are state-owned enterprises (excluding central enterprises), followed by non-state-owned enterprises, and central enterprises are the weakest.

This article’s potential contributions are as follows: First, it enriches the relevant research on ESG and government subsidies, constructs a framework system for the relationship between ESG and government subsidies, expands the research on the antecedents of government subsidies, improves the economic utility of ESG, increases its popularity, and provides an important starting point for alleviating corporate financing problems; Secondly, it reveals the internal mechanism by which ESG affects government subsidies, confirms that corporate transparency plays a positive mediating role, and further defines the logical relationship between the two, providing inspiration for enterprise management to put the ESG idea into practice; Thirdly, building on the practical context and based on political ties and property rights, further analyze the article’s conclusions. This will inspire management to use the ESG system to gain economic benefits and regulatory authorities to implement differentiated management, increasing the article’s practical value and significance.

## 2. Research background and literature review

In the 2021 global carbon dioxide emissions ranking, China ranked first with a proportion of 45%, the United States ranked second with a proportion of 20%, and other countries accounted for the remaining 35% of global carbon emissions. China’s carbon emissions level is close to nearly half of the global total carbon emissions. As the world’s second largest economy and the world’s largest industrial country, the traditional business philosophy of prioritizing economic benefits overlooks environmental issues. Some enterprises adopt excessive emissions of pollutants and excessive development of natural resources to maximize economic benefits. Therefore, solving China’s environmental problems urgently requires a new business philosophy. ESG, an indicator system that measures the development level of enterprises from three aspects: environment, society, and corporate governance, is in line with China’s environmental problems and provides an important direction for solving environmental problems. In June 2018, A-shares were officially included in the MSCI Emerging Markets Index, and the ESG evaluation system entered China. In 2020, the report of the 20th National Congress emphasized the harmonious coexistence between humans and nature, and attached great importance to environmental and ecological governance. According to the 《China ESG Development Report 2021》, in 2018, there were 872 A-share listed companies in China that released ESG related independent reports. In 2021, there were 1130 companies, and the number has been increasing year by year. In 2021, China released policy documents such as the 《Management Measures for Legal Disclosure of Enterprise Environmental Information》, which strengthened the country’s attention to ESG from the institutional level. Meanwhile, Chinese academic community has also conducted relevant research on ESG, mainly exploring the economic consequences of ESG. Previous studies have pointed out that improving ESG for enterprises can help alleviate financing constraints, reduce information, operational, and financial risks, improve business efficiency, and significantly reduce audit fees [11], thereby enhancing enterprise value [17]. To comprehensively showcase existing research results, this article also analyzes relevant literature from the United States and Europe. As the second largest country in total carbon emissions, the United States has also conducted research on the ESG system in the academic community. A study on listed companies in the United States found that ESG is beneficial for enhancing corporate value [14] and improving corporate performance [15]. ESG related research in the European region has pointed out that the more actively companies practice social responsibility, the less they participate in quick eye strategy, which can greatly reduce actual earnings management behavior [21]. Negative ESG performance can affect investors’ reactions, increase equity capital costs, and is not conducive to corporate financing [22]. For banks, ESG has a significant positive impact on bank performance [23].

Regarding research on government subsidies, existing literature in China points out that there is a positive correlation between corporate environmental responsibility and government subsidies received by enterprises [24]. Good performance of corporate social responsibility can not only reflect good business conditions and gain government trust, but also demonstrate a good image and strengthen political connections, thereby improving the level of government subsidies received [25]. Research in the United States has focused more on the impact of government subsidies on agriculture and industry, as well as federal subsidies to states. Regarding the literature on European government subsidies, existing studies have pointed out that renewable energy has gained significant public support through subsidies, and the withdrawal of subsidies will affect companies’ investment in European renewable energy [26]. And Europe has invested a lot of money in labor market policies [27].

The above analysis shows that existing research mainly explores the economic consequences of ESG, including aspects such as company performance and corporate value, and has not yet addressed the impact of government subsidies. The research on government subsidies has not yet delved deeply into the indicator system. In addition, existing research on ESG and government subsidies mainly adopts the OLS regression method. Based on the background of "dual carbon", this article studies the connection between ESG and government subsidies, bringing the relationship between government and enterprises closer, expanding the economic and political utility of ESG, enriching the research on the antecedents of government subsidies, and filling the gap in existing research.

## 3. Theoretical analysis and research assumptions

### 3.1 ESG performance and government subsidies

Government subsidies for the development of enterprises take the form of financial subsidies, tax incentives, technological innovation, and other aspects. Some scholars believe that actively fulfilling corporate social responsibility is beneficial for enhancing corporate image and strengthening political connections [28]. The emerging indicator system of ESG measures the performance of corporate social responsibility from three aspects: environment, society, and corporate governance. By disclosing the true business information of enterprises, it provides an important reference for the government to select subsidy recipients. The reasons why improving an enterprise’s ESG performance is beneficial for obtaining more government subsidies are mainly summarized in the following two aspects: First, good ESG performance means that enterprises have better fulfilled their obligations in environmental protection, social responsibility, and corporate governance, which is consistent with the corporate image required by the government to achieve public goals and conducive to improving the political connection of enterprises and increasing the possibility of obtaining government subsidies. Scholars have pointed out that enhancing corporate social responsibility can help enterprises get more government funding and favorable treatment [7]. Secondly, based on signal transmission theory, signals with strong credibility often have their own unique advantages that are difficult to imitate [29]. After effective investigation and verification by professional evaluation institutions, the ESG rating index can serve as a reliable signal to transmit information about the true business situation of enterprises to the outside world [9], alleviate the problem of information asymmetry between government and enterprises, and assist the government in obtaining and screening information prior to subsidies. A high ESG rating is a positive signal that a company has fulfilled its social responsibilities and has the potential for sustainable development, which is beneficial for attracting government attention and increasing the likelihood of enterprises receiving government subsidies.

In order to deeply explore the mechanism of ESG on government subsidies and further clarify the logical relationship between the two, this article conducts the following analysis and exploration: Due to the issue of information asymmetry between the government and firms in the process of defining subsidy targets, enterprises as information providers can utilize their information advantages to modify their external image and further obtain more resources. However, as a user of information, the government is often at a disadvantage and cannot fully understand the true business situation of enterprises, resulting in inefficient or inaccurate government selection of subsidy targets. As an important supplement to financial information, the ESG system can reflect the three aspects of environmental, social, and corporate governance performance, help to improve corporate transparency, disclose their operating conditions and sustainable development capabilities to a greater extent, and provide an important source of information for the government to make subsidy decisions. Therefore, this article explores the intermediary mechanism of ESG’s impact on government subsidies from the perspective of corporate transparency. The specific analysis can be summarized in the following three points.

Firstly, analyze from the perspective of agency costs. The management-shareholder relationship is a principal-agent relationship in which the management is entrusted with the management of the enterprise’s internal activities and decision-making matters, but there is information asymmetry and inconsistent interests between the two parties, with the shareholders pursuing the maximization of corporate interests and the management pursuing the maximization of personal interests [30]. Therefore, as the scale of the enterprise expands, conflicts between the two subjects often arise, leading to the generation of principal-agent costs. The efficiency of corporate governance is affected [31]. The ESG system can alleviate the principal-agent problem. Companies with good ESG performance tend to have sound internal governance mechanisms, sound management models, and efficient information transfer, which can help reduce the information barrier between shareholders and management, alleviate the agency problem [32], promote the convergence of shareholders’ and management’s philosophies, and improve decision execution efficiency. At the same time, enterprises with high ESG ratings pay attention to environmental risk management, and the management has a strong awareness of environmental protection. In terms of corporate decision-making, shareholders and management will make decisions and deployments based on long-term strategic goals, which is conducive to mitigating principal-agent risk and reducing agency costs [33]. The reduction of proxy costs greatly reduces corporate costs and expenses, which is conducive to promoting the effective allocation of resources, improving corporate transparency and the quality of external information disclosure [9], further attracting the attention of the government, and enhancing the possibility of obtaining government subsidies.

Secondly, analyze from the perspective of stakeholders. Referring to existing literature [34], according to the idea of "selecting winners", the government is more willing to select truly excellent enterprises, subsidize projects with high success rates, and minimize market distortion. Additionally, scholars have also pointed out that fulfilling corporate social responsibility is beneficial for improving stakeholder relationships and gaining more trust [35]. However, in reality, enterprises may engage in negative events that violate their original intentions of operation and harm social benefits driven by interests [36]. These enterprises often face condemnation and pressure from the external environment, ultimately bearing the negative consequences of damaged reputations and hindered development. On the contrary, enterprises that greatly fulfill their social responsibilities stand out in society and transmit positive signals about their good business performance to the outside world. This not only aligns with the government’s logical thinking of "selecting winners" from a rigid perspective and meets the government’s standards for target subsidy enterprises, but is also conducive to close relationships with the government and furthering government trust. The ESG system can comprehensively reflect the degree of corporate social responsibility fulfillment and comprehensively demonstrate the operational ability and sustainable development level of enterprises by improving corporate transparency [9]. A good ESG performance indicates that the company has a good balance of social and economic benefits, a sound internal governance mechanism, and a robust management model, which are in line with the government’s "winner criteria" and help the company stand out in the social competition and improve the relationship between the government and the company to a greater extent, thus helping to increase the level of government subsidies received by the company.

Thirdly, analyze from the perspective of reputation effects. A Good reputation can play a catalytic and buffering role, bringing positive economic benefits to enterprises while reducing the cost of releasing bad news. On the one hand, a positive reputation effect can act as a positive signal for a company to build its brand image, build reputation capital [37], attract greater attention from stakeholders, and thus increase the likelihood that the company will receive government subsidies. On the other hand, when an enterprise releases negative news, the reputation effect can play a buffering role, providing more time and opportunities for the enterprise to handle the situation and reducing financial difficulties and operational crises that may be caused by bad news [38]. The ESG system is consistent with China’s "dual carbon" and high-quality economic growth policies. It abandons the traditional profit-first business philosophy, advocates for enterprises to balance economic and social benefits, and comprehensively measures the degree of corporate social responsibility performance from the three dimensions of environment, society, and corporate governance. It can serve as a reliable signal to transmit the true operational information of enterprises to the outside world [9], improving corporate transparency. Good ESG performance provides a signal of sound corporate governance mechanisms, good business conditions, and strong development potential to the public, which can help enterprises establish a good brand image and reputation effect [39], gaining greater government attention and trust, and thereby improving the level of government subsidies received by enterprises. Based on the above analysis, the following research hypotheses are proposed:

Hypothesis H1: Controlling other conditions unchanged, companies can receive more government subsidies if they improve their ESG performance.

Hypothesis H2: Controlling other conditions unchanged, corporate transparency plays a positive mediating role in the process of companies improving their ESG performance and thus raising the level of government subsidies.

### 3.2 The perspective of political connection

Government subsidies are transfer payments provided by the government based on political and economic policies to achieve social governance goals. They are a sign of an enterprise’s positive reputation and prospects for growth. There is frequently a problem with information asymmetry between the government and businesses when deciding who should receive government subsidies [33]. Political connections can serve as a link between enterprises and the government, mitigating the impact of information asymmetry on enterprises, enabling enterprises to understand standards for subsidy recipients in advance, make timely preparations by regulating operational behavior, further enhancing the possibility of obtaining government subsidies. In contrast, enterprises without political connection are at an information disadvantage due to a lack of political advantages, lack opportunities to show the government their actual operational capabilities. The three components of the ESG system—environment, society, and corporate governance—are primarily used to communicate company information from a non-financial standpoint. As an important supplement to financial information, it can comprehensively reflect the fulfillment situation of corporate social responsibility and sustainable development capabilities. Enterprises with good ESG performance can increase government subsidies in the following three aspects: Firstly, by demonstrating the steady economic situation of the enterprise, enterprises with good ESG performance often have good financial conditions [40], sufficient cash flow, and considerable profitability. They also focus on increasing investment in R&D to enhance innovation capabilities, laying the foundation for obtaining long-term benefits [41]; Secondly, by showing the complete governance mechanism of the enterprise. Enterprises with high ESG ratings frequently have strong internal controls, efficient information flow, solid governance structures, management models, and operational systems, as well as low financial and non-systemic risks; Thirdly, by demonstrating the positive business philosophy of the enterprise, a high ESG rating indicates that the enterprise has a strong sense of social responsibility, a holistic perspective, and a good handle on balancing the benefits of the economy and society. It shows that the enterprise actively fulfills its social obligations and builds up moral advantages and reputation capital [42].

Based on the above analysis, a good ESG rating can reflect an enterprise’s stable operating condition and sustainable development ability from multiple aspects, which is conducive to breaking information barriers and winning over stakeholders. Therefore, enterprises without political connection are more motivated to increase their levels of government subsidies by improving ESG performance. As the tightness between government and enterprises increases, the accumulation of political resources will to some extent reduce the dependence of enterprises on the ESG system, thereby weakening the motivation of enterprises to obtain higher levels of government subsidies by improving ESG performance. Therefore, when political connections become stronger, enterprises become less inclined to increase government subsidies through improved ESG performance. In summary, the following research proposition is proposed:

Hypothesis H3a: Controlling other conditions unchanged, compared with companies with political connections, companies without political connections are more likely to improve their ESG performance thus increase government subsidies.

Hypothesis H3b: Controlling other conditions unchanged, the strength of political connections plays a surrogate role for companies to improve their ESG performance and thus increase government subsidies.

## 4. Research design

### 4.1 Sample selection and data source

This paper selects the 2011–2020 Huazheng corporate ESG score index to study the relationship between corporate ESG performance and government subsidies. In this paper, the data are processed as follows: (1) Eliminate financial enterprise samples; (2) Eliminate ST and PT enterprise samples; (3) Eliminate samples with missing data. At the same time, in order to reduce the interference of abnormal data on the regression results, we performed Winsorize processing on all continuous variables at the 1% and 99% quantiles, and the data in this paper were obtained through Stata17 processing statistics.

### 4.2 Variable definition

#### 4.2.1 Explained variable

The explained variable is government subsidies. Government subsidies can be measured in two ways. One way is to measure the absolute value of government subsidies (GOV), that is, the total amount of government subsidies received by enterprises in the year plus one to take the natural logarithm. Another way is to measure the relative value of government subsidies (GOV2), which is the ratio of government subsidies to total assets during the year. The larger value under two measurement methods, the higher level of government subsidies. Government subsidies come from other income accounts in income statement and government subsidy detail accounts in non-operating income.

#### 4.2.2 Core explanatory variable

The core explanatory variable is a company’s ESG performance. As a professional enterprise providing index services, China Securities has a certain authority and professionalism in the field of financial investment. China Securities ESG Index has the advantages of wide coverage and complete data, which is suitable for the research of Chinese market to be carried out, so this paper selects Huazheng ESG rating index to measure ESG performance of enterprises. Huazheng ESG rating is divided into 9 grades above AAA, AA, A, BBB, BB, B, CCC, CC, and C. For the convenience of data analysis and statistics, it is assigned as 9, 8, 7, 6, 5, 4, 3, 2, 1, the higher corporate ESG performance rating, the better corporate ESG performance, the stronger corporate social responsibility, and the stronger sustainable development capability.

#### 4.2.3 Mediating variable

Analysts and auditors, as important intermediaries of information, are closely related to corporate transparency, and earnings quality can reflect financial transparency. Referring to existing research [43, 44], this article uses five indicators to measure corporate transparency: earnings quality indicators, information disclosure score values, number of analysts tracking, analyst earnings prediction accuracy, and whether the enterprise hired the International Big Four as auditors for its annual report. Based on existing model [45], the earnings quality indicator is calculated, the larger indicator value, the higher the earnings quality. The information disclosure test scores are based on the information disclosure quality published by Shenzhen Stock Exchange in each year, and are divided into four grades: A, B, C, and D (excellent, good, pass, and fail). The scores are assigned 1, 2, 3, and 4 from low to high. The score is higher, the quality of information disclosure is higher. The number of analyst trackers refers to the number of analysts who forecast the company’s annual earnings in that year. The number is larger, the transparency is higher. The greater the accuracy of analyst earnings forecasts, the more accurate the forecasts and the greater the transparency. Auditor sub-index, the high rigor of the four major audited financial reports improves corporate transparency. The enterprise transparency index (TRANS) is obtained by taking the average of the sample percentage grades of the above five indicators. The value is larger, the transparency of an enterprise is higher. If any of the above indicators are missing, the percentile average of the remaining indicators is calculated to represent corporate transparency.

#### 4.2.4 Moderating variable

This paper studies the influence mechanism of corporate ESG performance on government subsidies from the political connection background and political connection strength. For different political connection backgrounds (PC), if any one of the chairman and general manager of the company is a current or former government official, the value is 1, otherwise it is 0. Referring to existing research [46], quantify the strength of political connection (PC Level). If the chairman or general manager of the company has or is currently serving in the government, party committee (disciplinary commission), the National People’s Congress or CPPCC permanent body, procuratorate and court, the PC Level will be assigned to four levels: the value of section-level cadres is 1, the value of department-level cadres is 2, the value of department-level cadres is 3, the value of minister-level cadres is 4, and the value of no political connection is 0. If the chairman or general manager of the company was or is currently serving as a party representative, a representative of the National People’s Congress or a member of the CPPCC, the PC Level will be assigned to four levels: the value of district and county level and below is 1, the value of municipal level is 2, the value of provincial level is 3, the value of national level is 4, and the value of no political connection is 0. If there are data in both level definition methods of PC Level, take the maximum value of the two definition methods as the value of the enterprise’s political connection level.

#### 4.2.5 Control variable

Referring to existing research [24], this paper selects the following indicators as control variables: return on total assets (ROA), which is the ratio of annual net profit to the average annual balance of total assets; financial leverage (LEV), which is the ratio of total liabilities at the end of the year to total assets at the end of the year; enterprise size (SIZE), which is the natural logarithm of total assets at the end of the period; enterprise age (AGE), which is the natural logarithm of the listing year plus one; institutional investor shareholding ratio (CI), which is the ratio of the number of shares held by institutional investors to the company’s total shares; the shareholding ratio of the largest shareholder (TOP1), which is the ratio of the number of shares held by the company’s largest shareholder to the company’s total shares; fixed assets ratio (CAP), which is the ratio of fixed assets to total assets at the end of the year; the concurrent position of the chairman and the general manager (DUAL), that is, the chairman and the general manager are the same person take 1, and different people take 0; board size (INBO), which is the natural logarithm of the number of board members. This paper controls for both year (YEAR) and industry (IND) fixed effects. The measurement definitions and detailed measurement methods of each variable are shown in Table 1.

**Table 1:** Definition and measurement of variables.

Variable type	Variable name	Variable symbol	Variable definition
Explained variable	Government subsidy	GOV	The total amount of government subsidies received by enterprises in the year plus one to take the natural logarithm.
		GOV2	Proportion of government subsidies to total assets within the year, the larger the ratio, the higher the government subsidy.
Core explanatory variable	ESG performance	ESG	According to the ESG rating results, it is divided into 9 grades (AAA, AA, A, BBB, BB, B, CCC, CC, C) and assigned as (9, 8, 7, 6, 5, 4, 3, 2, 1).
Mediating variable	Corporate transparency	TRANS	Take the average of the sample percentage grades of five variables including comprehensive earnings quality indicators, information disclosure test scores, the number of analyst trackers, the accuracy of analysts’ earnings forecasts, and whether the company hired the four major international auditors for its annual report.
Moderating variable	Political connection	PO	Political connection represents how closely a business is connected to the government.
			1. Political connection background (PC): 1 for politically connected enterprises, 0 otherwise.
			2. Strength of political connection (PC Level): The strength of corporate political connections is divided into 5 levels from low to high (0, 1, 2, 3, 4).
Control variable	Return on total assets	ROA	The ratio of annual net profit to the average annual balance of total assets
	Financial leverage	LEV	The ratio of total liabilities at the end of the year to total assets at the end of the year.
	Enterprise size	SIZE	The natural logarithm of total assets at the end of the period.
	Enterprise age	AGE	The natural logarithm of the listing year plus one.
	Institutional investor shareholding ratio	CI	The ratio of the number of shares held by institutional investors to the company’s total shares.
	The shareholding ratio of the largest shareholder	TOP1	The ratio of the number of shares held by the company’s largest shareholder to the company’s total shares.
	Fixed assets ratio	CAP	The ratio of fixed assets to total assets at the end of the year.
	The concurrent position of the chairman and the general manager	DUAL	If the chairman and the general manager are the same person, take 1, if they are different, take 0.
	Board size	INBO	The natural logarithm of the number of board members.
	Year dummy variable	YEAR	In the current year, take 1, otherwise take 0.
	Industry dummy variable	IND	In this industry, take 1, otherwise take 0.

### 4.3 Model setting

In order to verify the hypothesis H1, the model (1) is set for empirical testing, in which GOV, it is the government subsidy, and ESG, it is the ESG performance of the enterprise. If the coefficient of β1 is significantly positive, the hypothesis H1 is established. In order to test the hypothesis H2, this paper sets up models (2)-(3) to form a mediation effect test model. If the sign of (γ1×θ1) is positive, it is a positive mediation effect, otherwise, it is a negative mediation effect. If (γ1×θ1) with the same sign as β1, it is a general mediating effect, otherwise it is a “Masking effect”. In order to verify the hypothesis H3a, this paper sets the model (4) to perform group regression on the political connection background (PC). If the non-political connection group α2 is significantly positive and the political connection group α2 is not significant, then the hypothesis H3a is established. Model (5) is a moderating effect model used to test the hypothesis H3b. ESGPC Level is the multiplication term of ESG and PC Level. If α3 is significantly negative, hypothesis H3b is established. This paper uses OLS (Ordinary Least Squared) Regression. The optimal linear unbiased estimates under classical statistical assumptions are generated based on the principle of least squares by adding series of relevant variables, controlling for industry and year, as a way to investigate the linear relationship between the independent and dependent variables and to verify the research hypotheses of this paper. Due to the OLS estimation method being able to minimize the sum of squares of the differences between the observed dependent variable and the predicted dependent variable, which minimizes the error rate between prediction and practice, reduces prediction losses, and improves prediction accuracy, this article chooses the OLS regression method for empirical analysis.


$$GOV_{i,t}=\beta_{0}+\beta_{1}ESG_{i,t}+\beta \sum C\mathrm{on}trols_{i,t}+YEAR+IND+\varepsilon_{i,t}$$ (1)

$$TRANS_{i,t}=\gamma_{0}+\gamma_{1}ESG_{i,t}+\gamma \sum C{\mathrm{ontrols}}_{i,t}+YEAR+IND+\varepsilon_{i,t}$$ (2)

$$GOV_{i,t}=\theta_{0}+\theta_{1}TRANS_{i,t}+\theta_{2}ESG_{i,t}+\theta \sum Controls_{i,t}+YEAR+IND+\varepsilon_{i,t}$$ (3)

$$GOV_{i,t}=\alpha_{0}+\alpha_{1}ESG_{i,t}+\alpha_{2}PC+\alpha \sum Controls_{i,t}+YEAR+IND+\varepsilon_{i,t}$$ (4)

$$\begin{array}{c} GOV_{i,t}=\alpha_{0}+\alpha_{1}ESG_{i,t}+\alpha_{2}PCLevel_{i,t}+\alpha_{3}ESGPCLevel_{i,t}\\ +\alpha \sum Controls_{i,t}+YEAR+IND+\varepsilon_{i,t} \end{array}$$ (5)

## 5. Analysis of empirical results

### 5.1 Descriptive statistics

Table 2 lists the descriptive statistics about each variable. The maximum value of government subsidy (GOV) is 20.25, minimum value is 0, standard deviation is 3.401, the mean is 15.77, and the median is 16.34. The above data shows that there are large differences in the government subsidies received by various enterprises. The maximum value of ESG performance (ESG) is 9, the minimum value is 1, the standard deviation is 1.152, the mean is 6.582, and the median is 6, which is highly consistent with existing research [47]. The above data shows that the ESG performance of many companies is at a moderate level, and the ESG performance of different companies varies greatly. Regarding the control variables, most of the sample firms have low financial leverage (LEV), firm size (SIZE), shareholding of the first largest shareholder (TOP1), and fixed assets ratio (CAP), while most of the sample firms have a high level of return on total assets (ROA), firm age (AGE) and board size (INBO). The shareholding of institutional investors (CI) in the sample firms is more evenly distributed, and most of the sample firms have non-identical chairmen and general managers.

**Table 2:** Descriptive statistics.

Variable	Sample size	Mean	Standard deviation	Minimum	Median	Maximum
GOV	19500	15.77	3.401	0	16.34	20.25
ESG	19500	6.582	1.152	1	6	9
ROA	19500	0.0357	0.0555	-0.209	0.0331	0.187
LEV	19500	0.454	0.203	0.0647	0.451	0.894
SIZE	19500	22.42	1.296	19.92	22.25	26.33
AGE	19500	2.317	0.736	0.693	2.485	3.258
CI	19500	0.425	0.230	0.00158	0.438	0.872
TOP1	19500	35.78	15.03	9.534	33.85	74.89
CAP	19500	0.227	0.167	0.00162	0.195	0.711
INBO	19500	2.148	0.196	1.609	2.197	2.708
DUAL	19500	0.235	0.424	0	0	1

### 5.2 Correlation analysis

Table 3 shows the correlation analysis results of each variable. It can be seen from the table that the correlation coefficient between corporate ESG performance (ESG) and government subsidy (GOV) is 0.080, which is significant at the 1% level. From this, it can be concluded that there is a significant positive correlation between corporate ESG performance and government subsidy. There is a significant positive correlation at the 1% level between GOV and ROA, LEV, SIZE, CI, TOP1, CAP, and INBO. There is a significant negative correlation between GOV and AGE. The correlation coefficients between all variables in Table 3 are all less than 0.5, and further VIF test shows that the VIF value of each variable is less than 5, indicating that there is no serious multicollinearity among the variables.

**Table 3:** Correlation analysis results.

Panel A											
Variable	GOV	ESG	ROA	LEV	SIZE	AGE	CI	TOP1	CAP	INBO	DUAL
GOV	1										
ESG	0.080***	1									
ROA	0.061***	0.134***	1								
LEV	0.066***	0.095***	-0.365***	1							
SIZE	0.312***	0.368***	0.014**	0.486***	1						
AGE	-0.048***	0.157***	-0.184***	0.301***	0.308***	1					
CI	0.113***	0.264***	0.098***	0.175***	0.425***	0.284***	1				
TOP1	0.063***	0.136***	0.137***	0.043***	0.218***	-0.119***	0.409***	1			
CAP	0.123***	-0.015**	-0.075***	0.033***	0.064***	0.038***	0.081***	0.064***	1		
INBO	0.058***	0.142***	0.0100	0.132***	0.234***	0.109***	0.181***	0.017**	0.144***	1	
DUAL	-0.00100	-0.110***	0.032***	-0.115***	-0.153***	-0.224***	-0.177***	-0.053***	-0.081***	-0.182***	1
Panel B											
VIF test	ESG	ROA	LEV	SIZE	AGE	CI	TOP1	CAP	INBO	DUAL	Mean
	1.21	1.29	1.63	1.81	1.35	1.57	1.33	1.04	1.12	1.10	1.35

Note: *** P<0.01

** P<0.05

* P<0.1.

### 5.3 Analysis of basic regression results

Table 4 shows the regression results of ESG on government subsidies, in which column (1) does not control for year (YEAR) and industry (IND), without adding control variables. The results show that the regression coefficient of ESG is 0.237, which is significant at the 1% level. Column (2) controls for year (YEAR) and industry (IND) and adds the control variables. The regression results show that the regression coefficient of ESG is 0.059, which is also significantly positive at the 1% level. The adjusted R2 in column (2) is 0.267, compared to 0.006 in column (1), which indicates that the regression model fits better with the inclusion of control variables. The regression coefficient of ESG in column (2) decreases compared to column (1) after the inclusion of control variables, but it is still significant, indicating that the improvement of ESG performance of firms can enhance the level of government subsidies, and hypothesis H1 is verified.

**Table 4:** The impact of improved corporate ESG performance on government subsidies.

	(1)	(2)
Variable	GOV	GOV
ESG	0.237***	0.059***
	(11.32)	(3.35)
ROA		1.211***
		(2.62)
LEV		0.320*
		(1.84)
SIZE		1.079***
		(36.49)
AGE		-0.559***
		(-16.43)
CI		0.360***
		(3.30)
TOP1		-0.002
		(-1.06)
CAP		1.383***
		(7.45)
INBO		-0.151
		(-1.25)
DUAL		0.004
		(0.08)
Constant	14.208***	-7.613***
	(104.09)	(-11.82)
Year fixed effect	Uncontrolled	Controlled
Industry fixed effect	Uncontrolled	Controlled
Sample size	19500	19500
Adjust R2	0.006	0.267

Note: *** P<0.01

** P<0.05

* P<0.1.

### 5.4 The perspective of corporate transparency

Table 5 shows the results of the mediation effect test of corporate transparency. The regression results of columns (1) and (2) control for year (YEAR) and industry (IND). The regression coefficient of ESG in column (1) is 0.019, which is significantly positive at the 1% level. The regression coefficient of TRANS in column (2) is 0.424, which is also significantly positive at the 1% level. The sign of (γ1×θ1) is positive consistent with β1, indicating that corporate transparency exerts a general mediating effect, not a “masking effect”, suppose H2 holds, that is, corporate transparency plays a positive mediating effect in the process of companies improving their ESG performance and thus increasing the level of government subsidies.

**Table 5:** The mediation effect test of corporate transparency.

	(1)	(2)
Variable	TRANS	GOV
TRANS		0.424***
		(2.86)
ESG	0.019***	0.051***
	(18.70)	(2.89)
ROA	1.103***	0.743
	(50.80)	(1.43)
LEV	-0.072***	0.351**
	(-10.49)	(2.01)
SIZE	0.065***	1.051***
	(54.68)	(35.20)
AGE	-0.027***	-0.547***
	(-14.83)	(-16.16)
CI	0.135***	0.302***
	(23.47)	(2.73)
TOP1	-0.001***	-0.001
	(-10.37)	(-0.85)
CAP	0.004	1.381***
	(0.49)	(7.44)
INBO	-0.000	-0.151
	(-0.02)	(-1.25)
DUAL	0.009***	0.000
	(3.59)	(0.00)
Constant	-1.201***	-7.103***
	(-49.26)	(-11.04)
Year fixed effect	Controlled	Controlled
Industry fixed effect	Controlled	Controlled
Sample size	19500	19500
Adjust R2	0.426	0.267

Note: *** P<0.01

** P<0.05

* P<0.1.

### 5.5 The perspective of political connection

#### 5.5.1 The perspective of political connection background

Table 6 shows the results of the moderating effect test of political connections. The degree of an enterprise’s political connection affects the strength of an enterprise’s incentive to increase its government subsidies through improving ESG performance. Enterprises with strong political connections have the political advantage of being closely connected with the government and are more likely to obtain government subsidies, while enterprises without political connections need to use other channels to obtain government attention. The ESG system can act as an effective way for enterprises to obtain government subsidies by virtue of its authority and objectivity. In this paper, the sample is divided into two groups according to whether the enterprise has political connection. Column (1) of Table 6 is the group with political connection background, which is not statistically significant, and column (2) is the group without political connection background. The ESG coefficient is 0.088, which is significant at the 1% level. The comparison shows that enterprises with no political connection background are more inclined to increase their government subsidies by improving their ESG performance, assuming that H3a is established.

**Table 6:** Moderating effect test of political connection.

	(1)	(2)	(3)
Variable	Whether there is a political connection background		Strength of political connection
	Yes	No	
ESG	-0.000	0.088***	0.092***
	(-0.00)	(4.32)	(4.44)
ESGPCLevel			-0.034***
			(-2.79)
PCLevel			0.236***
			(3.02)
ROA	3.744***	0.119	1.225***
	(4.22)	(0.22)	(2.64)
LEV	1.355***	-0.165	0.332*
	(4.24)	(-0.81)	(1.91)
SIZE	0.919***	1.143***	1.078***
	(17.19)	(33.09)	(36.56)
AGE	-0.519***	-0.579***	-0.556***
	(-7.71)	(-14.99)	(-16.34)
CI	0.296	0.412***	0.361***
	(1.51)	(3.16)	(3.30)
TOP1	-0.003	-0.002	-0.002
	(-0.88)	(-1.11)	(-1.07)
CAP	1.009***	1.564***	1.387***
	(2.75)	(7.50)	(7.49)
INBO	-0.006	-0.292**	-0.150
	(-0.03)	(-2.02)	(-1.24)
DUAL	-0.056	0.022	0.011
	(-0.68)	(0.39)	(0.24)
Constant	-4.433***	-8.797***	-7.841***
	(-4.03)	(-11.21)	(-12.15)
Year fixed effect	Controlled	Controlled	Controlled
Industry fixed effect	Controlled	Controlled	Controlled
Sample size	6435	13065	19500
Adjust R2	0.253	0.291	0.267

Note: *** P<0.01

** P<0.05

* P<0.1.

#### 5.5.2 The perspective of the strength of political connection

Columns (1) and (2) of Table 6 show that for politically connected companies, the incentive to obtain more government subsidies by improving their ESG performance is weaker. Conversely, enterprises that are not politically connected have a stronger incentive to increase government subsidies by improving their ESG performance. In reality, the distribution of the intensity of political connections between different enterprises is different, and extreme cases do not represent the full picture of the company. This paper quantifies the intensity of political connection of enterprises from low to high. Column (3) of Table 6 shows that the coefficient of interaction between ESG and political connection strength is -0.034, which is significantly negative at the 1% level, indicating the influence of political connection strength and ESG performance on government subsidies is mutually replaceable, that is, as the strength of corporate political connections increases, the impact of improved ESG performance on the increase in government subsidies is weakened. Hypothesis H3b holds.

## 6. Robustness test

### 6.1 PSM test

High returns on assets, high liabilities, large scale, long listing years and other characteristics may promote enterprises to improve their ESG performance. In order to mitigate the impact of sample selection errors on the conclusion, referring to existing research [47], this paper divides the sample enterprises into two groups with the median of ESG as the boundary, and selects nine indicators of return on total assets (ROA), financial leverage (LEV), enterprise size (SIZE), enterprise age (AGE), institutional investor shareholding ratio (CI), the shareholding ratio of the largest shareholder (TOP1), fixed assets ratio (CAP), the concurrent position of the chairman and the general manager (DUAL), and board size (INBO). The logit model regression was constructed to obtain the predictive value, that is, the propensity score represents the overall level of interfering factors. The nearest neighbor matching method was used to find individuals with the same or similar characteristics for each individual in the treatment group from the control group as the control, and the paired samples were regression analyzed. As shown in column (1) of Table 7, the regression coefficient of ESG is 0.091, which is significant at the 1% level. This verifies that improving the ESG performance of enterprises can still increase the level of government subsidies again.

**Table 7:** Robustness test.

	(1)	(2)	(3)	(4)	(5)
Variable	PSM	Treatment effect model	GOV2	GOVt+1	Drop 2015 and 2020
ESG	0.091***	0.073**	0.000***	0.000***	0.063***
	(3.78)	(2.25)	(5.27)	(5.27)	(3.00)
IMR		-0.025			
		(-0.54)			
ROA	0.361	1.180**	0.004***	0.004***	1.425***
	(0.56)	(2.54)	(5.33)	(5.33)	(2.58)
LEV	0.201	0.328*	0.002***	0.002***	0.419**
	(0.85)	(1.87)	(6.33)	(6.33)	(2.06)
SIZE	1.106***	1.074***	-0.001***	-0.001***	1.102***
	(25.86)	(34.76)	(-15.71)	(-15.71)	(32.30)
AGE	-0.618***	-0.560***	-0.000***	-0.000***	-0.610***
	(-13.23)	(-16.44)	(-7.42)	(-7.42)	(-15.45)
CI	0.448***	0.354***	0.001***	0.001***	0.377***
	(3.14)	(3.24)	(6.03)	(6.03)	(2.93)
TOP1	-0.006***	-0.002	-0.000*	-0.000*	-0.004*
	(-2.73)	(-1.06)	(-1.66)	(-1.66)	(-1.94)
CAP	1.045***	1.382***	0.002***	0.002***	1.440***
	(4.12)	(7.44)	(8.92)	(8.92)	(6.68)
INBO	-0.015	-0.153	-0.000	-0.000	-0.194
	(-0.09)	(-1.27)	(-0.02)	(-0.02)	(-1.38)
DUAL	-0.149**	0.005	0.000	0.000	0.033
	(-2.25)	(0.10)	(0.47)	(0.47)	(0.61)
Constant	-8.187***	-7.598***	0.017***	0.017***	-7.880***
	(-8.51)	(-11.76)	(19.63)	(19.63)	(-10.72)
Year fixed effect	Controlled	Controlled	Controlled	Controlled	Controlled
Industry fixed effect	Controlled	Controlled	Controlled	Controlled	Controlled
Sample size	8981	19498	19500	19500	15372
Adjust R2	0.281	0.267	0.105	0.105	0.260

Note: *** P<0.01

** P<0.05

* P<0.1.

### 6.2 Treatment effect model test

Since self-selection may lead to enterprises obtaining government subsidies and improving ESG performance, and there may be omitted variables in the main regression model that may cause endogeneity problems, this paper uses a treatment effect model to alleviate endogeneity problems.

First, construct a selection equation for improving ESG performance, namely model (6).


$$ESGS_{i,t}=\eta_{0}+\eta_{1}IV_{i,t}+\eta Controls_{i,t}+YEAR+IND+\varepsilon_{i,t}$$ (6)

In the above formula, ESGS is a dummy variable that divides the enterprise samples into two groups according to the median ESG performance. IV is an instrumental variable, referring to existing research [48], the mean value of ESG performance of other firms in the same year and industry as the instrumental variable (IV) is selected, and this variable satisfies the requirements of relevance and exogeneity. Since the business environment characteristics of the same industry are largely the same, the ESG performance of the firm in that year will be influenced by the ESG performance of other firms in the same industry, which satisfies the condition of relevance, but the ESG performance of other firms at the industry level is derived based on macro analysis and will not have a direct impact on the level of government subsidies of the firm at the micro level, which satisfies the condition of exogeneity. Model (6) uses Probit regression and calculates the Inverse Mills Ratio (IMR). At the same time, due to hidden collinearity, two observations were eliminated after Probit regression, and the sample size was reduced to 19498.

Second, the inverse Mills ratio estimated by model (2) is added to model (1) to construct model (7). The results are shown in column (2) of Table 7. The coefficient of ESG is 0.073, which is significant at the 5% level, indicating that after considering the influence of the sample self-selection bias, the conclusion of this paper is still established, that is, the improvement of ESG performance of firms can enhance the level of government subsidies.


$$GOV_{i,t}=\eta_{0}+\eta_{1}ESG_{i,t}+\eta_{2}IMR_{i,t}+\eta Controls_{i,t}+YEAR+IND+\varepsilon_{i,t}$$ (7)

### 6.3 Replace explained variable

In order to verify the reliability of the finding that the improvement of ESG performance is conducive to the improvement of government subsidies, this paper changes the measure of the explained variables from the total amount of government subsidies received by enterprises in a year plus one by taking the natural logarithm (GOV) to the ratio of government subsidies to total assets in a year (GOV2).

The adoption of the ratio of government subsidies to total assets (GOV2) during the year achieves a shift from direct to indirect measurement and enhances the objectivity of government subsidy values. the total amount of government subsidies received by enterprises in the year plus one to Since the direct measurement method is susceptible to the influence of the general economic operation level of the country, and the social environment, international trade, and political stability are all linked to the economic situation of the country, the direct measurement method cannot objectively reflect the level of government subsidies to a certain extent, while the objective measurement method of the ratio of government subsidies to total assets during the year, excludes the interference of the general economic operation of the country and directly reflects the intensity of the level of government subsidies through the size of the ratio.The larger the value indicates a higher level of government subsidies. The regression coefficient of ESG on GOV2 is shown in column (3) of Table 7. The regression coefficient of ESG is significantly positive at the 1% level, which again validates the finding that improved ESG performance of firms is conducive to higher levels of government subsidies.

### 6.4 Use the explained variables of the t+1 period

In order to reduce the interference of the inter-causal endogeneity problem on the conclusions of this paper, this paper adopts a fixed-effect model that controls the year and industry, and selects the government subsidy in the (t+1) period as the explained variable for robustness test. The result is shown in column (4) of Table 7, the ESG coefficient is significantly positive at the 1% level, indicating that the improvement of corporate ESG performance will significantly increase future government subsidies, eliminate possible endogenous problems and improve the robustness of the conclusions of this paper.

### 6.5 Change the selection of sample year

The “stock market crash” in 2015 and the “new crown epidemic” in 2020 ravaged the world. The world economy suffered depression and turmoil under external shocks. The severe and cruel living environment affected the normal profitability and innovation development of enterprises. The strategic goals and business model of enterprises are severely challenged by global changes. In addition, ESG, an emerging indicator system for measuring the level of sustainable development of enterprises, has been in development in China not long and is easily affected by changes of the external environment. Therefore, the sample data selected in this paper may have abnormal values. In order to further improve the robustness of the conclusion, this paper removes the samples in 2015 and 2020 for regression analysis. The conclusion is shown in column (5) of Table 7. The regression coefficient of ESG is 0.063, which is significantly positive at the 1% level. It shows that under excluding influences of the stock market crash and the epidemic, the improvement of ESG performance can still increase government subsidies, which further strengthens robustness of the conclusions of this paper.

## 7. Further analysis

Enterprises with different property rights have significant differences in management models, business concepts, political connections, and other aspects. State-owned enterprises are the lifeblood of the national economy and dominate and play a leading role in key areas. Central enterprises are state-owned enterprises supervised and managed by the central government, as leaders among state-owned enterprises, they occupy a dominant position in major industries and key fields related to national security and the lifeline of the national economy. In view of the special property rights of state-owned enterprises and central enterprises, this paper further studies the impact of ESG performance on government subsidies based on the property rights heterogeneity of sample companies. First, this paper divides the sample of enterprises into three groups: non-state-owned enterprises, state-owned enterprises (excluding central enterprises), and central enterprises, and analyze the annual government subsidies in units of million received by different enterprise groups (GOVP). Further, the following models are constructed to regress, and the property rights variables are introduced, including whether it is a state-owned enterprise (SOE) and whether it is a central enterprise (YQ). If the enterprise is a state-owned enterprise, SOE is taken as 1, otherwise, it takes 0, if the enterprise is a central enterprise, YQ takes 1, otherwise it takes 0. The first regression is based on model (8), and on the premise of removing central enterprises, add ESG performance (ESG) and state-owned enterprise (SOE) interaction term ESGSOE to examine the influence of property rights heterogeneity between non-state-owned enterprises and state-owned enterprises (excluding central enterprises) on the conclusion; The second and third regressions are based on model (9), and carried out under the conditions of removing state-owned enterprises that are not central enterprises and removing central enterprises respectively, add ESG performance (ESG) and whether it is a central enterprise (YQ) interaction term ESGYQ to test the impact of property rights heterogeneity of two groups including non-state-owned enterprises and central enterprises, state-owned enterprises (excluding central enterprises) and central enterprises on the conclusions.


$$GOV_{i.t}=\lambda_{1}ESGSOE_{i,t}+\lambda_{2}ESG_{i,t}+\lambda_{3}SOE_{i,t}+\lambda Controls_{i,t}+YEAR+IND+\varepsilon_{i,t}$$ (8)

$$GOV_{i,t}=\lambda_{1}ESGYQ_{i,t}+\lambda_{2}ESG_{i,t}+\lambda_{3}YQ_{i,t}+\lambda Controls_{i,t}+YEAR+IND+\varepsilon_{i,t}$$ (9)

### 7.1 Property rights group analysis

It can be seen from the Table 8 that average annual government subsidies received by the non-state-owned enterprise group was 33.18 million yuan, the average annual government subsidies received by the state-owned enterprise (excluding central enterprises) group was 44.70 million yuan, and average annual government subsidy received by the central enterprise group was 80.98 million yuan. By comparing data between above groups, it can be seen that gap between non-state-owned enterprises and state-owned enterprises (excluding central enterprises) is not obvious, indicating that state-owned enterprises (excluding central enterprises) have little advantage in obtaining government subsidies. While the average annual government subsidy received by central enterprises compared to the other two groups has a significant gap, indicating that central enterprises have the advantage of obtaining government subsidies.

**Table 8:** Average value of government subsidies received by various enterprises in the year (million).

Variable	The non-state-owned enterprise	The state-owned enterprise (excluding central enterprises)	The central enterprise
GOVP	33.18	44.70	80.98

### 7.2 Heterogeneity analysis of property rights

Based on the property rights grouping analysis in Table 8, this paper further studies the impact of property rights heterogeneity on the relationship between ESG performance and government subsidies. The regression results are shown in Table 9. Column (1) of Table 9 shows the regression results of non-state-owned enterprises and state-owned enterprises (excluding central enterprises). The regression coefficient of ESGSOE is 0.130, which is significant positive. From the analysis in Table 8, we can see that level of government subsidies received by state-owned enterprises (excluding central enterprises) have no obvious advantages over non-state-owned enterprises, and the political advantages of state-owned enterprises make it easier to obtain information related to government subsidies. The political sensitivity of state-owned enterprises promotes enterprises to closely follow the national strategic layout and respond to the call of national policies. Therefore, state-owned enterprises (excluding central enterprises) have stronger incentives than non-state-owned enterprises to increase the level of government subsidies by improving ESG performance. Column (2) of Table 9 shows the regression results of non-state-owned enterprises and central enterprises. The regression coefficient of ESGYQ is -0.143, which is significant at the 5% level. Column (3) of Table 9 shows the regression results of state-owned enterprises (excluding central enterprises) and central enterprises. The regression coefficient of ESGYQ is -0.280, which is significant at the 1% level. The regression results of columns (2) and (3) correspond to the analysis results in Table 8. Since central enterprises are directly under the jurisdiction of the State Council, they have a significant advantage in easily obtaining government subsidies. Therefore, compared with other enterprises, central enterprises have weaker incentives to increase government subsidies by improving their ESG performance.

**Table 9:** Heterogeneity analysis of property rights.

	(1)	(2)	(3)
Variable	The non-state-owned enterprises and the state-owned enterprises (excluding central enterprises)	The non-state-owned enterprises and the central enterprises	The state-owned enterprises (excluding central enterprises) and the central enterprises
ESG	0.030	0.057***	0.315***
	(1.51)	(2.80)	(4.50)
ESGSOE	0.130*		
	(1.81)		
SOE	-0.783		
	(-1.54)		
ESGYQ		-0.143**	-0.280***
		(-2.40)	(-3.16)
YQ		1.025**	1.843***
		(2.53)	(2.96)
ROA	1.169**	1.206**	0.598
	(2.38)	(2.51)	(0.54)
LEV	0.181	0.183	1.379***
	(0.95)	(1.03)	(3.56)
SIZE	1.158***	1.102***	0.821***
	(33.90)	(36.14)	(16.93)
AGE	-0.622***	-0.574***	-0.316***
	(-16.97)	(-16.09)	(-3.43)
CI	0.303***	0.293***	0.930***
	(2.61)	(2.63)	(2.83)
TOP1	-0.003*	-0.001	-0.006
	(-1.79)	(-0.36)	(-1.39)
CAP	1.732***	1.599***	-0.450
	(8.43)	(8.41)	(-1.26)
INBO	-0.135	-0.045	-0.686**
	(-1.05)	(-0.36)	(-2.45)
DUAL	0.005	0.032	-0.028
	(0.09)	(0.68)	(-0.16)
Constant	-8.951***	-8.148***	-4.563***
	(-12.19)	(-12.37)	(-3.21)
Year fixed effect	Controlled	Controlled	Controlled
Industry fixed effect	Controlled	Controlled	Controlled
Sample size	17076	18093	3831
Adjust R2	0.266	0.273	0.275

Note: *** P<0.01

** P<0.05

* P<0.1.

## 8. Conclusion

With the deployment of the "dual carbon" strategy and high-quality economic development goals, green protection and sustainable development have become the themes of today’s enterprise development. ESG highly aligns with the economic background of China’s green transformation of enterprises, and evaluates the degree of corporate social responsibility fulfillment and sustainable development ability from three aspects: environment, society, and corporate governance, which is highly concerned by all sectors of society. The article empirically investigates the roles and impact mechanism of ESG on government subsidies using observational data from A-share listed businesses in Shanghai and Shenzhen from 2011 to 2020. Research has confirmed that improving ESG performance for enterprises is beneficial for obtaining more government subsidies, and has been further validated through robustness tests such as PSM test, treatment effect model test, replace explained variable, use the explained variables of the t+1 period and change the selection of sample year. In the mediation mechanism test, this article further found that corporate transparency plays a positive mediating role, clarifying the logical framework between ESG and government subsidies. Given the high political relevance of government subsidies, this article conducts in-depth research on political connections. The results show that non politically connected enterprises pay more attention to improving the level of government subsidies through good ESG performance, and the strength of political connections plays a substitute role in improving the ESG performance of enterprises and thereby increasing the level of government subsidies. Due to the close correlation between property rights and political connections, this article further explores the conclusions of property rights heterogeneity. The research results show that enterprises with different property rights have different strengths of motivation to increase government subsidies by improving ESG performance. State owned enterprises (excluding central enterprises) are the strongest, followed by non-state-owned enterprises, and central enterprises are the weakest. The above content enriches existing research on ESG and government subsidies from a new research perspective, expands the economic utility of ESG, provides effective inspiration for alleviating financing constraints of enterprises, promotes sustainable development of enterprises and healthy competition in the capital market, and points out key directions for regulatory departments to supervise enterprises’ "washing green" behavior, which is conducive to improving the accuracy of government support and effective resource allocation. It has management significance and practical value.

Based on the above conclusions, the following implications for management are proposed:

Firstly, promote the popularity of the ESG system. ESG is in line with China’s present policy environment, which is conducive to promoting business sustainability as well as economic transformation and upgrading. At the same time, due to the fact that the number of years of ESG prevalence in China is still insufficient, the positive economic effects have not been fully realized. Therefore, the Chinese government and enterprises should work together to promote the popularity of ESG. The government should actively incorporate the ESG concept into the top-level design, increase ESG system publicity, establish and improve ESG-related laws and regulations, and improve the ESG information disclosure system. Nowadays, given the significant benefits of the ESG system for enterprises sustainable development, some companies have resorted to "greenwashing" behavior to whitewash ESG performance and falsely promote it in order to create a good reputation externally and obtain high-quality resources at the lowest possible cost. Therefore, the government should integrate ESG into the corporate credit system, increase supervision of "greenwashing" behavior, eliminate the phenomenon of enterprises using ESG to seek illegal private interests, and maintain a fair and healthy market competition order. As the evaluation subject of ESG, enterprises should actively practice ESG concepts, balance economic and social benefits. Management should incorporate ESG concepts into corporate culture, management mechanisms, and development goals, actively disclose the true operating status of enterprises to the public, improve information transparency, and create a good external image and reputation effect by transmitting positive signals to the outside world, enhance competitiveness in a legal and compliant manner and promote the good operation of the capital market.

Secondly, enterprises with different political connections ought to use differentiated management. By examining the moderating effect of political connection, this article confirms that non-politically affiliated enterprises pay more attention to improving government subsidy levels through good ESG performance, and the impact of political connection and ESG performance on government subsidies is mutually complementary. This finding provides good insight for companies to make up for the lack of political connections and alleviate financing problems. For enterprises with low political connections, management should strengthen attention to environmental protection, social responsibility, and corporate governance. They should also adopt green and energy-saving measures, strengthen R&D and innovation investment to reduce environmental protection costs, enhance corporate social responsibility and overall perspective through charitable donations, and further transform internal governance structures, business concepts, and management models to strengthen corporate governance. By using an ESG disclosure system, businesses can openly communicate information about their successful operating environments, attracting the attention of a wide range of stakeholders, gaining greater trust, and alleviating the financing pressure on enterprises. Enterprises that have strong political connections may experience the phenomenon of exploiting political expediency to secure relatively high government subsidies. In reality, there are situations where some enterprises use strong political connections to obtain more government subsidies and then abuse them, seriously wasting social resources and affecting fair distribution of resources and smooth operation of the capital market. Therefore, government should strengthen supervision of enterprise information acquisition, background investigation, and business capability evaluation, track the application fields, utilization efficiency, and achievements of subsidies in the process of resource distribution, dynamically adjust the amount of subsidies received by enterprises, improve the fairness of government subsidy distribution, promote improvement in resource utilization efficiency, and help further achieve social goals.

Thirdly, differentiated management should be implemented for enterprises with different property rights. This article has confirmed that enterprises with different property rights have different strengths of motivation to increase government subsidies by improving their environmental, social, and governance (ESG) performance. State-owned enterprises (excluding central enterprises) are the strongest, followed by non-state-owned enterprises, and central enterprises are the weakest. The above conclusions provide financing insights for the transformation and upgrading of enterprises with different property rights, which is conducive to fully leveraging the powerful engine role of ESG. In reality, there are phenomena where some enterprises rely on their political advantages to obtain sufficient political resources and achieve rent-seeking, as well as situations where zombie enterprises still occupy social resources due to the special nature of property rights. The above negative phenomena are not conducive to fair market competition or the healthy operation of the economy. Therefore, for the government, differentiated regulatory policies should be formulated based on the heterogeneity of property rights, and investigations into the operating status, background, and profitability of enterprises should be increased to effectively identify the true and false components of ESG performance, strengthen supervision of the subsequent application of government subsidies, timely rectify low-quality enterprises, eliminate zombie enterprises, improve national resource utilization efficiency, and achieve mutual benefit and win-win between the government and enterprises. At the same time, in order to further alleviate the information asymmetry between government and enterprises, enterprises can propose the establishment of an ESG committee to express their interests and demands, promote efficient communication between the committee and government, push the maximization of the economic effectiveness of ESG, and achieve enterprise transformation and upgrading.

## Supporting information

S1 Data (XLS)
